# Enrichment-based DNA methylation analysis using next-generation sequencing: sample exclusion, estimating changes in global methylation, and the contribution of replicate lanes

**DOI:** 10.1186/1471-2164-13-S8-S6

**Published:** 2012-12-17

**Authors:** Michael P Trimarchi, Mark Murphy, David Frankhouser, Benjamin AT Rodriguez, John Curfman, Guido Marcucci, Pearlly Yan, Ralf Bundschuh

**Affiliations:** 1 Comprehensive Cancer Center, The Ohio State University, Columbus, Ohio, USA; 2 Departments of Physics and Biochemistry, The Ohio State University, Columbus, Ohio, USA

## Abstract

**Background:**

DNA methylation is an important epigenetic mark and dysregulation of DNA methylation is associated with many diseases including cancer. Advances in next-generation sequencing now allow unbiased methylome profiling of entire patient cohorts, greatly facilitating biomarker discovery and presenting new opportunities to understand the biological mechanisms by which changes in methylation contribute to disease. Enrichment-based sequencing assays such as MethylCap-seq are a cost effective solution for genome-wide determination of methylation status, but the technical reliability of methylation reconstruction from raw sequencing data has not been well characterized.

**Methods:**

We analyze three MethylCap-seq data sets and perform two different analyses to assess data quality. First, we investigate how data quality is affected by excluding samples that do not meet quality control cutoff requirements. Second, we consider the effect of additional reads on enrichment score, saturation, and coverage. Lastly, we verify a method for the determination of the global amount of methylation from MethylCap-seq data by comparing to a spiked-in control DNA of known methylation status.

**Results:**

We show that rejection of samples based on our quality control parameters leads to a significant improvement of methylation calling. Additional reads beyond ~13 million unique aligned reads improved coverage, modestly improved saturation, and did not impact enrichment score. Lastly, we find that a global methylation indicator calculated from MethylCap-seq data correlates well with the global methylation level of a sample as obtained from a spike-in DNA of known methylation level.

**Conclusions:**

We show that with appropriate quality control MethylCap-seq is a reliable tool, suitable for cohorts of hundreds of patients, that provides reproducible methylation information on a feature by feature basis as well as information about the global level of methylation.

## Background

The promise of personalized medicine is that each patient receives customized treatment from a broad base of options rather than a single, generalized standard of care treatment [1]. This is especially important in cancer where each patient's cancer could be viewed as a separate disease caused by a unique set of aberrations. The rapidly decreasing cost of Next Generation Sequencing (NGS) is rendering this personalized approach a reality. For diseases with relatively high treatment costs, such as cancer, it is now economically viable to obtain whole genome sequencing data for the affected individual as part of the treatment regimen, and with further decreases in cost more and more diseases will follow suit.

However, the genomic sequence of malignant cells only partially captures the abnormalities that lead to malignancy. Other factors such as gene expression levels and epigenetic signals have to be taken into account when characterizing a specific cancer and deciding on an individual's treatment regimen. One prominent epigenetic signal for which a dysregulation in various types of cancer is already well established [2] is the addition of methyl groups at the 5' carbon of cytosine nucleotides [3,4].

There are several different methods to obtain genome-wide methylation information using NGS. The most reliable method is bisulfite conversion, where the genomic DNA is treated with sodium bisulfite to convert unmethylated cytosines into uracils and subsequently thymines upon PCR amplification [5]. Sequencing of the converted DNA immediately reveals the degree of methylation at any genomic cytosine by counting the number of observed cytosines vs. thymines; however complete methylome profiling using this method requires sequencing depths far beyond what is feasible today on the scale of larger patient cohorts. The sequencing depth requirements can be significantly alleviated by focusing coverage in CpG-rich genomic regions (e.g., using reduced representation bisulfite sequencing [6]), but this comes at the expense of greatly diminished genomic-wide coverage. The method used in our lab, MethylCap-seq [7], instead uses the methyl-binding domain of human MBD2 in order to enrich fragmented genomic DNA based on methylation content. Sequencing the fragments bound to the MBD2 domain provides a genome-wide view of methylation patterns at reasonable sequencing depths.

While the cost aspect of MethylCap-seq is attractive, it has two limitations. First, resolution is at the level of the DNA fragment size, i.e., about 150bp, rather than at the level of the individual CpG. This is not that problematic as long as one is only interested in characterizing the methylation status of extended genomic regions such as CpG islands, promoters, non-coding RNAs, or gene bodies. Second, the number of reads covering a genomic region is only a relative indicator of the amount of methylation in this region, relative to the sample genome as a whole, and thus data normalization is required to compare methylation between samples. This somewhat indirect nature of methylation status determination makes this method prone to data quality issues stemming from poorly prepared libraries. Also, somewhat paradoxically, one of the first parameters one might be interested in knowing, namely the degree of overall methylation of the sample, cannot be directly extracted from the data since relative methylation is encoded in the relative number of reads covering different genomic regions, yet the total number of reads is fixed by the sequencing itself rather than by the actual level of overall methylation in the sample. Here, we first perform a systematic study of the influence of sample quality and the contribution of additional reads (beyond ~13 million unique aligned reads) in MethylCap-seq data. Then, we show experimental evidence that a computational approach for determining overall methylation levels from MethylCap-seq data we recently suggested [8] approximates actual overall methylation levels. These studies underpin the usability of MethylCap-seq as a reliable method to obtain genome-wide methylation information at reasonable cost.

## Methods

### Patient samples

Tissue samples from an endometrial cohort including tumors from 89 endometrial patients and 12 nonmalignant endometrial samples were obtained from Washington University. All studies involving human endometrial cancer samples were approved by the Human Studies Committee at the Washington University and at The Ohio State University.

A subset of 7 ovarian cancer samples from a larger cohort was obtained from TriService General Hospital, Taipei, Taiwan. All studies involving human ovarian cancer samples were approved by the Institutional Review Boards of TriService General Hospital and National Defense Medical Center.

A subset of 14 bone marrow samples from a single-center Phase II trial of patients with acute myeloid leukemia (AML) at The Ohio State University was obtained for this investigation. The study design and the results of the trial for the entire cohort of patients have been reported elsewhere [9]. All studies involving these samples were approved by The Ohio State University Human Studies Committee.

### Methylated-DNA capture (MethylCap-seq)

Enrichment of methylated DNA was performed with the Methyl Miner kit (Invitrogen) according to the manufacturer's protocol as previously described [10]. Briefly, one microgram of sonicated DNA was incubated at room temperature on a rotator mixer in a solution containing 3.5 micrograms of MBD-Biotin Protein coupled to M-280 Streptavidin Dynabeads. Non-captured DNA was removed by collecting beads with bound methylated DNA on a magnetic stand and washing three times with Bind/Wash Buffer. Enriched, methylated DNA was eluted from the bead complex with 1M NaCl and purified by ethanol precipitation. Library generation and 36-bp single-ended sequencing were performed on the Illumina Genome Analyzer IIx according to the manufacturer's standard protocol.

### MethylCap-seq experimental quality control and exclusion criteria

The automated quality control (QC) module was implemented as previously described [10]. Pre-aligned sorted.txt files from the Illumina CASAVA 1.7 pipeline were utilized in the interest of quick turnaround for our users. In brief, duplicate alignments were removed from the aligned sequencing file (a correction for potential PCR artefacts), and the resulting output was loaded into an R workspace. MEDIPS [11] was utilized to perform CpG enrichment, saturation, and CpG coverage analyses.

Sequencing lanes were identified for exclusion using the following thresholds: CpG enrichment < 1.4, saturation < 0.5, CpG 5x coverage < 0.05. These criteria and corresponding thresholds were chosen based on their technical relevance and ability to stratify datasets with known technical issues without a salient bias towards biological groups. Samples were excluded if any of the thresholds were not met. As CpG coverage was assessed qualitatively for analysis of the Endometrial dataset, five lanes of data with borderline 5x CpG coverage were not excluded that would have qualified for exclusion due to this criterion.

For the DMR comparison (Table 1), methylation signal was normalized for each lane and then averaged among replicate lanes for each sample. The "All" group thus contains samples with merged QC pass lanes, samples with merged QC fail lanes, and samples with merged QC pass and QC fail lanes.

For the reproducibility comparison (Additional file 1), Pearson r was calculated using 2 replicate lanes corresponding to each sample represented in the QC pass and QC fail groups. In the case that a sample had more than two replicate lanes in a single group, two lanes were randomly chosen for the analysis. Samples lacking two replicate lanes in either the QC pass or QC fail group were excluded from this analysis. Lanes corresponding to the same sample but generated using different library preparations were also excluded.

We routinely provide sequencing and QC summaries for our users, and the summaries corresponding to the datasets referenced in this manuscript can be viewed in Additional files 2, 3, and 4.

### Standard sequence file processing and alignment

Sequence files were processed and aligned as previously described [10]. Briefly, QSEQ files from the Illumina CASAVA1.7 pipeline were converted to FASTA format, duplicate reads removed (to control for PCR bias), and then uniquely aligned with Bowtie to generate SAM files using the following options: -f -t -p 1 -n 3 -l 32 -k 1 -m 1 -S -y --chunkmbs 1024 -max -best [12]. Duplicate alignments (reads aligning to the same genomic position) were removed using SAMtools [13].

### Standard global methylation analysis workflow

Aligned sequence files in SAM format were analyzed using our custom analysis workflow as previously described [10]. Briefly, aligned reads were extended to the average fragment length (as determined by BioAnalyzer fragment analysis) and counted in 500 bp bins genome-wide. The resulting count distribution was normalized against the total aligned reads by conversion to reads per million (RPM). These normalized genome-wide count files were then interrogated by genomic feature (e.g., CpG islands, CpG shores, promoters). Differentially methylated regions were identified by summing RPM across the bins for each locus in the genomic feature, then performing a Wilcoxon rank sum test to assess differences in these summed RPMs between sample groups. Results were then adjusted for multiple comparisons by setting a false discovery rate (FDR) cutoff of 0.05.

### Calculation of noise in methylation signal

Noise in methylation signal, representing extended reads falling in regions without CG dinucleotides, was quantified as the summation of reads falling into bins with zero CpG content. In the case that a sample in a given group had multiple lanes of data, noise was computed for each lane individually and averaged among replicate lanes in the group. As a single sample could have a lane that passed QC and a lane that failed QC, the number of samples in each group does not sum to the total number of samples in the study.

### Calculation of the Global Methylation Indicator

To assess genome-wide changes in methylation patterns for each sample across a given experiment, a custom parameter termed the global methylation indicator (GMI) was calculated as previously described [8]. Briefly, normalized read counts (in RPM) were classified by CpG density and averaged to construct a methylation distribution. The average RPM were then summed across the distribution (i.e., the estimated area under the methylation distribution curve) to yield the GMI.

### Assessment of methylated fragment enrichment using an *in vitro *methylated construct

#### Experimental procedure

The 5.3 kb plasmid vector pIRES2-EGFP, which contains three CpG islands, was chosen to empirically assess methylated fragment enrichment. The construct was linearized with Nhe I and then *in vitro *methylated with M.*Sss*I. The methylated spike-in DNA was quantified by Qubit high sensitivity assay and diluted. Plasmid was spiked into genomic DNA at a concentration of 1.5 pg plasmid/1 μg genomic DNA (~2.5 plasmid copies per cell) prior to sonication of genomic DNA for library generation.

#### Analysis

Reads mapping to the construct were identified by converting QSEQ files to FASTA format as described above, then aligning the files with Bowtie using the following options: -q -t -p 1 -n 3 -l 32 -k 1 -S --chunkmbs 1024 --max --best. Duplicate reads were retained for this analysis. To control for variation in construct aligned read counts that might be attributable to fluctuations in lane yield, construct aligned read counts were normalized against the total raw read counts by conversion to reads per million (RPM).

## Results and discussion

### Quality control exclusion criteria reduce noise in methylation signal and improve analytical power

Our automated quality control (QC) module, which is based on MEDIPS [11], was implemented to identify technical problems in the sequencing data and flag potentially spurious samples. One goal of the QC module was to provide rapid feedback to investigators regarding dataset quality, facilitating protocol optimization prior to committing resources to a larger scale sequencing project. A second goal was to identify samples that should be excluded from analyses due to data validity concerns. The validity of a MethylCap-seq experiment is dependent on enrichment of methylated fragments prior to sequencing. A failure in enrichment invalidates any downstream data, and therefore identifying such failures is vital. Also important is verifying the statistical reproducibility of the data for each sample. As it is often not cost-effective to generate replicate sequencing lanes for each sample to assess experimental reproducibility empirically, addressing this issue computationally is desirable. Similarly, the confidence in methylation calls is related to the breadth and strength of signal at the CpGs in the genome. We assessed enrichment of methylated fragments using the CpG enrichment parameter, which compares the frequency of CpGs in the sequenced sample with the frequency of CpGs in the reference genome. Statistical reproducibility was assessed by calculation of saturation, the Pearson correlation of two random partitions of the sequenced sample [11]. Breadth and strength of methylation signal was assessed using 5X CpG coverage, which represents the fraction of CpG loci that have five or more reads in the sample compared to the total number of CpGs in the reference genome. These QC parameters were calculated for each sample using MEDIPS [11].

**Figure 1:**
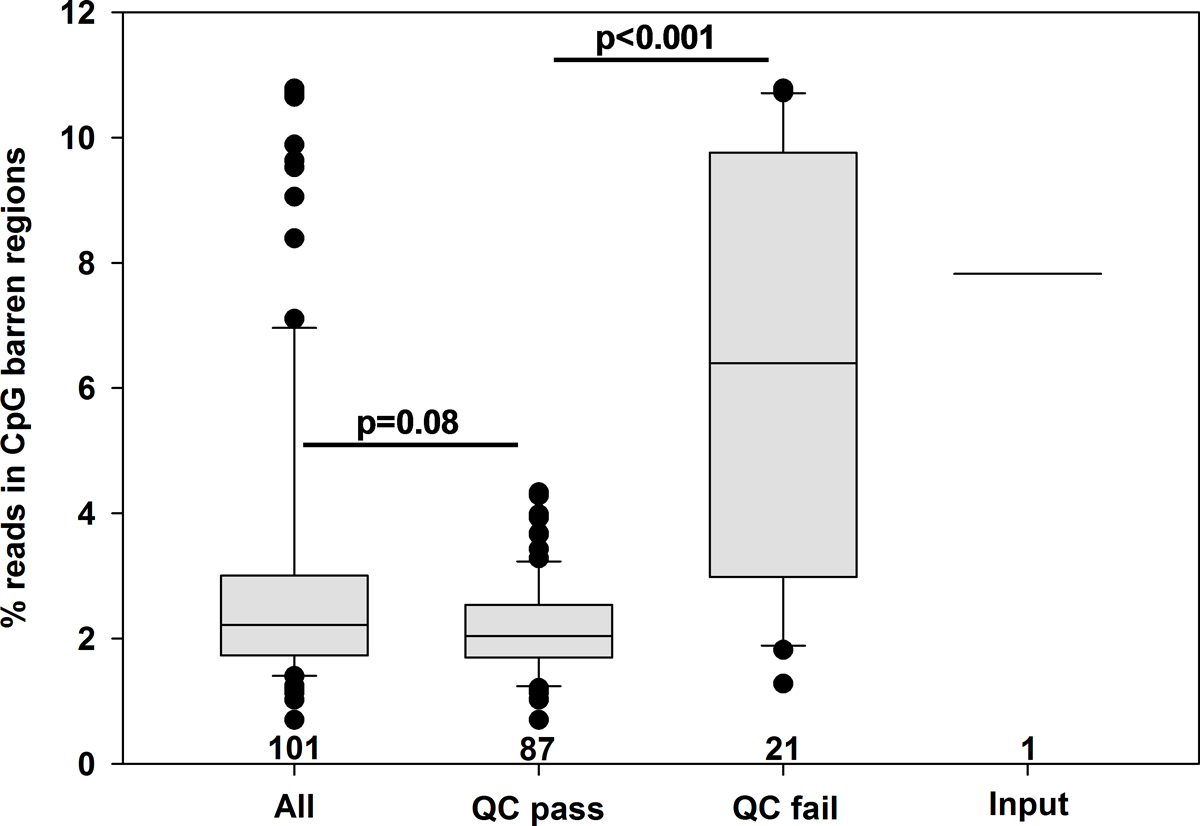
**QC exclusion criteria reduce noise in methylation signal**. The percentage of uniquely aligned reads falling in 500 bp bins containing no CpG dinucleotides pre- and post-QC analysis are plotted as a standard boxplot for samples prior to QC filtering, samples that passed QC, and samples that did not pass QC. An input from a sample that was not subjected to methylation capture is included for reference. The number of samples in each group is included above the baseline. Values for replicate lanes in each group were averaged, and samples were statistically compared using a Wilcoxon rank-sum test. Whiskers indicate 10^th ^and 90^th ^percentiles. 13.5% of 500 bp bins in the genome are classified as CpG barren.

Additional file 2 demonstrates the results of the QC module for the Endometrial dataset. 203 lanes of sequencing data were generated for 101 unique samples. 43 lanes failed QC, representing 21 unique samples. To assess how lanes that pass QC might differ from lanes that failed QC, we computed the noise in methylation signal, representing percentage of uniquely aligned extended reads falling in 500 bp bins without CpG dinucleotides (Figure 1). Median noise in samples that failed QC (6.40%) was more than 3-fold greater than in samples that did not fail QC (2.04%, p < 0.001), and closely resembled noise in input (7.82%). Excluding QC failed lanes did not significantly decrease median noise levels (2.04 vs. 2.22, p = 0.08), but did greatly decrease the variation in noise levels between samples. As the distribution of noise levels is positively skewed and not normal, a small number of outliers would not be expected to significantly shift the median noise level. To investigate whether the additional noise seen in QC failed samples impacted sequencing reproducibility, we computed the Pearson correlation between replicate lanes of samples that passed QC vs. failed QC (Additional file 1). Replicates of samples that passed QC correlated much more highly than replicates of samples that failed QC (average r = 0.90 vs. 0.59; p < 0.001). Variation in replicate correlation between samples was also noticeably less in the QC pass group (relative standard deviation = 6.7% vs. 27.1%). We surmise that failures in methylation enrichment result in a more random sampling of the fragment distribution regardless of methylation status, resulting in increased signal in regions where methylation should not be detectable.

As the goal of many methylome profiling studies is to identify differentially methylated regions (DMRs) between biological groups, we next assessed whether our QC exclusion criteria might improve our analytical power to detect DMRs. We compared DMRs between 89 endometrial tumors and 12 nonmalignant endometrial tissue samples across several genomic features. Excluding sequencing lanes that failed QC (corresponding to 19 tumor and 2 nonmalignant samples) resulted in more DMRs in every genomic feature assessed (Table 1). The greatest gains were seen in promoters and CpG shores, where the number of DMRs increased 22-fold and 2-fold, respectively, while gains in CpG islands and promoter-associated CpG islands were more modest (1.6-fold and 1.05-fold). These results appear to trend inversely with CpG density, possibly reflecting greater benefit from QC exclusion in regions where coverage is lower. We speculate that the improvements in DMR detection resulting from exclusion of samples that fail QC would be even greater when working with smaller sample sizes or biological groups with more similar methylation patterns.

**Table 1:** Differentially methylated regions, endometrial tumors vs. nonmalignant endometrial tissue

Genomic feature	All samples	Samples passing QC only
CpG islands	4717	7541
Promoter- associated	3806	3980
CpG shores	7515	15371
Promoters	314	6803

### The effect of additional sequencing lanes on quality control metrics

**Figure 2:**
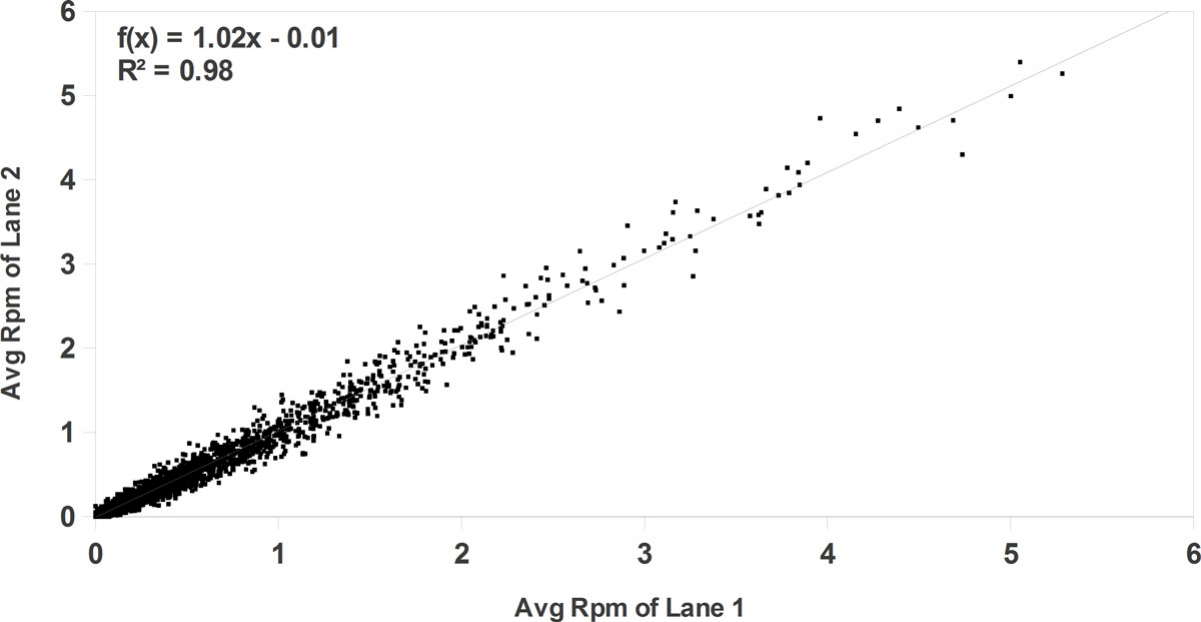
**Replicate sequencing lanes for MethylCap-seq experiments correlate highly**. Replicate lanes for each sample were randomly assigned to two partitions, and the average rpm of 6000 (of 6 M) randomly selected 500 bp bins were compared between partitions.

**Figure 3:**
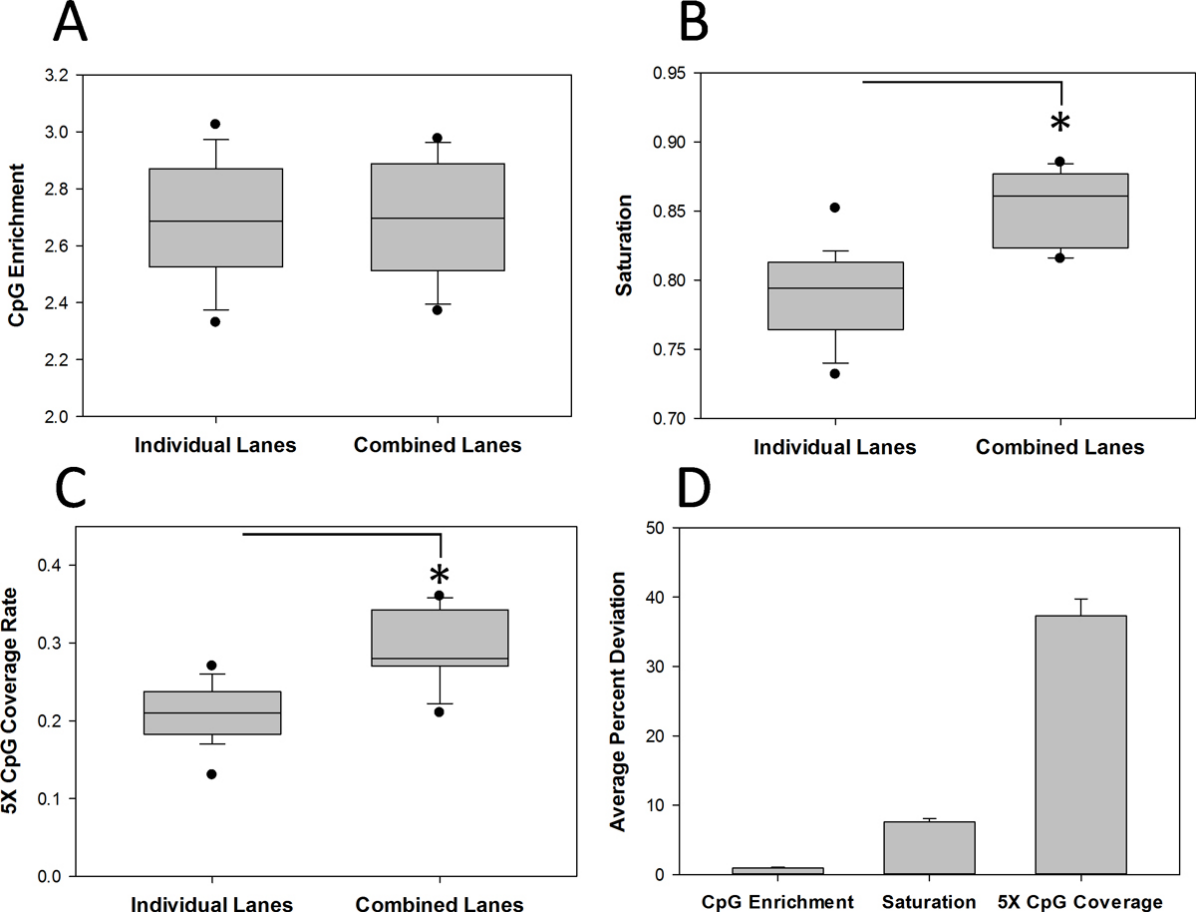
**Additional lanes of sequencing data moderately increase saturation and greatly increase 5X CpG coverage**. Variation in CpG enrichment (A), saturation (B), and 5X coverage (C) was assessed for 15 lanes of data in the ovarian study corresponding to 7 samples by generating plots of individual lanes and combined replicate lanes for each sample. (D) Average percent deviation of the individual lanes from the combined lane for each sample was plotted for each parameter. Error bars for (D) represent standard error. Asterisks represent Student t-test p < 0.05.

As a sequencing core, we are frequently asked whether additional lanes of sequencing data are necessary or desirable for MethylCap-seq experiments. To address this issue, we analyzed a large dataset of ovarian tumors, of which 7 samples had been resequenced (using the same genomic library), for a total of 15 lanes (Additional file 3). Before comparing the effect of additional lanes, the degree of correlation between the replicate lanes was analyzed to ensure that additional lanes of data would not introduce excessive variation. As shown in Figure 2, replicate lanes from sequencing the same library twice correlated highly (R^2 ^value of 0.98; note, that we here specifically address the question of the value of additional sequencing lanes and not of additional technical or biological replicates - the correlation between technical or biological replicates would be expected to be much lower than the correlation between two lanes sequencing the same library shown here). CpG enrichment, saturation, and 5X coverage were then evaluated for individual lanes and combined lanes (Figure 3). CpG enrichment varied somewhat between samples (range: 2.33-3.02), but was extremely similar for replicate lanes ( < 1% percent deviation from the combined lane on average). Saturation improved modestly from a median of 0.79 to a median of 0.86. As saturation values for individual lanes of MethylCap-seq data typically range from 0.6 to 0.85 for single lanes in our hands, and we consider a saturation value of 0.6 acceptable for analysis, this improvement may be inconsequential although it is statistically significant. 5X coverage improved noticeably from a median of 0.21 to a median of 0.28, representing an average 38% gain. As 5X coverage represents a minimum signal level needed to reliably differentiate a methylated locus from a locus with no methylation (or the absence of a methylation signal), we speculate that this increase could significantly increase the statistical power to detect DMRs, particularly in small or lightly methylated regions.

### Global methylation indicator correlates inversely with an *in vitro *methylated indicator sequence

We recently proposed a computational method to compare genome-wide changes in methylation patterns between samples in a given experiment [8]). As MethylCap-seq signal (in reads) is normalized by total aligned read counts to adjust for variability in lane yield, two samples with identically distributed methylation yet different absolute levels of methylation would be expected to yield identical normalized methylation signals at any given loci. The GMI method relies on the observation that *in vitro *methylated samples display characteristic changes in the methylation signal distribution as quantified in a MethylCap-seq experiment, and these changes are CpG density dependent. Methylation signal shifts from low CpG content regions to high CpG content regions, and this can be quantified by calculating the area under the curve of the average normalized methylation signal plotted across CpG density. The GMI calculation is a potentially powerful tool for capturing changes in global methylation between samples.

**Figure 4:**
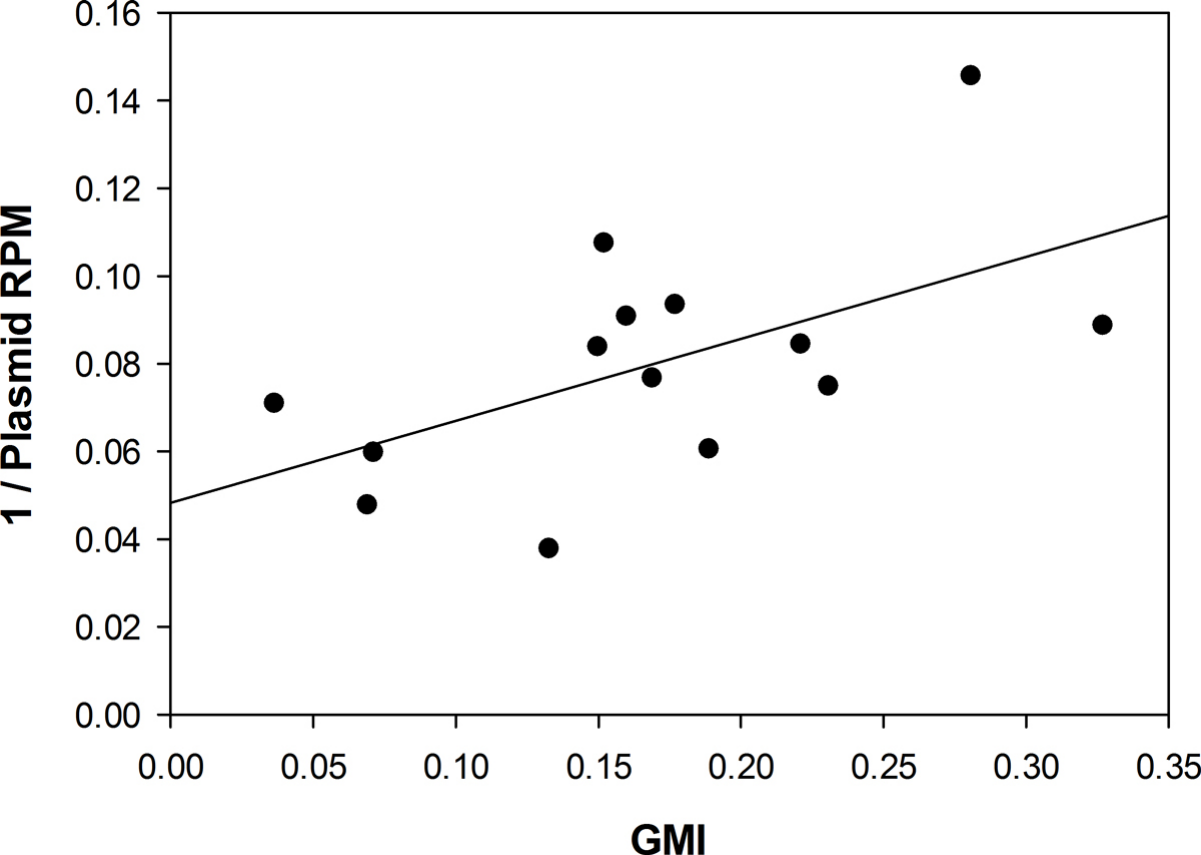
**Global methylation indicator scales inversely with read counts from a spiked-in *in vitro *methylated construct**. The pIRES2-EGFP plasmid was *in vitro *methylated and spiked-in at a set concentration into each of 14 samples from the decitabine study prior to sequencing. After sequencing, GMI was calculated and plotted against the inverse of the number of normalized reads aligning to the plasmid, and a linear best fit drawn through the points (p = 0.036, R^2 ^= 0.318).

In an effort to validate the GMI as a surrogate for global methylation, we developed a complementary analysis utilizing an *in vitro *methylated construct. This methylated construct was spiked-in to the genomic DNA in the AML samples prior to sonication at a defined concentration and subjected to methylated enrichment along with the genomic DNA. The spike-in was originally intended to verify successful enrichment; if enrichment occurred, PCR for the methylated plasmid would show increased copy number after enrichment. However, this spike-in is also a way to determine global methylation levels since the methylated plasmid competes with the natively methylated genomic DNA fragments for binding to the MBD protein. When the proportion of methylated to unmethylated genomic fragments is high prior to enrichment, the methylated plasmid gets enriched relatively less, and vice versa. Indeed, we found that read counts aligned to the plasmid correlate inversely with GMI (Figure 4, Additional file 4). This result provides empirical evidence that GMI can capture changes in absolute global methylation levels for MethylCap-seq experiments. Such a metric might be useful for gauging response to treatments that are known or expected to globally alter the methylome.

## Conclusions

We show that post-sequencing QC metrics can be used to exclude poor quality samples from analysis, resulting in decreased noise in methylation signal and improved power to detect DMRs. Furthermore, we show that resequenced lanes from the same library correlate very well, and that additional lanes of data have a small impact on saturation (data reproducibility) and a large impact on 5X CpG coverage (confidence in methylation calls at a given locus). Finally, we demonstrate that our computational indicator of global methylation correlates with an unrelated method that utilizes spike-in of DNA with known methylation status. These findings verify that with appropriate quality control MethylCap-seq is a reliable tool that provides reproducible relative methylation information on a feature by feature basis, provides information about the global level of methylation, and can be applied to entire patient cohorts of hundreds of patients.

## List of abbreviations used

NGS: Next-generation sequencing; AML: acute myeloid leukemia; QC: quality control; RPM: reads per million; FDR: false discovery rate; GMI: global methylation indicator; DMR: differentially methylated region.

## Competing interests

The authors declare that they have no competing interests.

## Authors' contributions

MT, MM, DF, RB, and PY designed the study. MT and RB drafted the manuscript. MT, MM, DF, and BR analyzed the data. RB, PY, and BR critically reviewed the manuscript. PY, BR, and JC generated the data. GM procured samples. RB, PY, and GM oversaw the project.

## Supplementary Material

Additional file 1 **Replicate lane correlation, endometrial QC passed vs. QC failed samples**. A table showing Pearson correlation of replicate lanes for samples that passed QC vs. failed QC. Data is presented both as a group summary and for individual samples. Click here for file

Additional file 2 **QC table for endometrial cancer study**. A listing of CpG enrichment, saturation, 5x coverage, and read information for each sample lane in the endometrial dataset. Click here for file

Additional file 3 **QC table for ovarian study**. A listing of CpG enrichment, saturation, 5x coverage, and read information for each sample lane in the ovarian dataset. Click here for file

Additional file 4 **QC, GMI, plasmid RPM table for AML study**. A listing of CpG enrichment, saturation, 5x coverage, read information, global methylation indicator, and plasmid reads per million for each sample lane in the AML dataset. Click here for file
